# Hypofractionated high-energy proton-beam irradiation is an alternative treatment for WHO grade I meningiomas

**DOI:** 10.1007/s00701-017-3352-4

**Published:** 2017-10-24

**Authors:** Pavlos Vlachogiannis, Olafur Gudjonsson, Anders Montelius, Erik Grusell, Ulf Isacsson, Kristina Nilsson, Erik Blomquist

**Affiliations:** 1 Department of Neuroscience, Neurosurgery, Uppsala University, Uppsala University Hospital, 751 85 Uppsala, Sweden; 2 Department of Immunology, Genetics and Pathology, Medical Radiation Science, Uppsala University Hospital, 751 85 Uppsala, Sweden; 3 Department of Immunology, Genetics and Pathology, Experimental and Clinical Oncology, Uppsala University, Uppsala University Hospital, 751 85 Uppsala, Sweden

**Keywords:** Meningioma, Benign meningioma, Proton beam irradiation, Hypofractionation

## Abstract

**Background:**

Radiation treatment is commonly employed in the treatment of meningiomas. The aim of this study was to evaluate the effectiveness and safety of hypofractionated high-energy proton therapy as adjuvant or primary treatment for WHO grade I meningiomas.

**Method:**

A total of 170 patients who received irradiation with protons for grade I meningiomas between 1994 and 2007 were included in the study. The majority of the tumours were located at the skull base (*n* = 155). Eighty-four patients were treated post subtotal resection, 42 at tumour relapse and 44 with upfront radiotherapy after diagnosis based on the typical radiological image. Irradiation was given in a hypofractionated fashion (3–8 fractions, usually 5 or 6 Gy) with a mean dose of 21.9 Gy (range, 14–46 Gy). All patients were planned for follow-up with clinical controls and magnetic resonance imaging scans at 6 months and 1, 2, 3, 5, 7 and 10 years after treatment. The median follow-up time was 84 months. Age, gender, tumour location, Simpson resection grade and target volume were assessed as possible prognostic factors for post-irradiation tumour progression and radiation related complications.

**Results:**

The actuarial 5- and 10-year progression-free survival rates were 93% and 85% respectively. Overall mortality rate was 13.5%, while disease-specific mortality was 1.7% (3/170 patients). Older patients and patients with tumours located in the middle cranial fossa had a lower risk for tumour progression. Radiation-related complications were seen in 16 patients (9.4%), with pituitary insufficiency being the most common. Tumour location in the anterior cranial fossa was the only factor that significantly increased the risk of complications.

**Conclusions:**

Hypofractionated proton-beam radiation therapy may be used particularly in the treatment of larger World Health Organisation grade I meningiomas not amenable to total surgical resection. Treatment is associated with high rates of long-term tumour growth control and acceptable risk for complications.

## Introduction

Meningiomas are benign tumours, accounting for about 14–37% of primary intracranial tumours [8, 17, 29]. Total surgical resection of the meningioma and its associated dural base is generally the treatment of choice, since it results in long-term disease-free survival in the majority of patients [4, 39, 43]. However, not all skull base meningiomas can be totally resected without an unacceptable risk of postoperative neurological deficits, particularly cranial nerve palsies [6, 16, 27]. Further treatment is needed for patients with postoperative residual tumour masses, recurrent tumours, multiple tumours, or primarily inextirpable tumours [24, 35].

There is compiling evidence indicating that radiotherapy has a valuable role in patients suffering from both benign and atypical meningiomas. This is in spite of a lack of well-performed randomised studies [19]. Both shrinkage of existing tumour burden and prevention of regrowth are possible gains [26, 31]. In retrospective studies conventionally fractionated radiotherapy has been reported to have an effect. Common schedules utilised have generally been 1.8– to 2.0-Gy fractions 5 days/week to a total dose of 50–60 Gy [2, 5, 14, 40]. Stereotactic radiosurgery using higher fraction doses have also been reported to play a role in the treatment of meningiomas. Most authors have utilised single fractions in the range of 15–25 Gy given either with the Leksell Gamma Knife® or with Linear Accelerators (LINAC) equipped with stereotactic instrumentation [7, 9, 10, 21, 34, 38]. In general, reported times of follow-up have been rather short in these studies and in series of meningiomas treated with proton radiation [15, 45, 47].

The dose distribution of a high-energy proton beam facilitates irradiation with a high dose to the target and comparatively low dose to the surrounding normal tissue due to the proton beam’s innate ability of conforming to the target by losing a large amount of energy at the end of the beam path (the Bragg peak) [11, 32]. A typical dose plan with modified proton beams given from two portals results in full dose coverage of a benign meningioma. No secondary photons or electrons are delivered downstream. The integral dose is lower compared to most photon techniques. A complicating factor with proton therapy seems to be the varying value of relative biological effectiveness (RBE). The total dose, number of fractions and type of tissue may affect the value, as pointed out by Bleddyn Jones [19]. RBE is not known for brain tissue, the optical apparatus and brainstem. However a factor of 1.1 has been accepted for treatment protocols at most proton therapy centres [32].

In the present study, we evaluated proton beam irradiation for the treatment of benign meningiomas in relation to clinical benefit and possible side effects. The aim was to find out if stereotactic irradiation with protons inhibits regrowth of residual tumour or progress of inextirpable meningiomas without causing undesirable effects on the brain.

## Materials and methods

### Patient population

A total of 183 patients underwent hypofractionated proton-beam radiation therapy as adjuvant or primary treatment for benign meningiomas between 1994 and 2007 at the "The Svedberg Laboratory" in Uppsala, Sweden. Nine of these patients were found to have a diverging pathologoanatomic diagnosis (PAD), that is other than World Health Organisation (WHO) grade I meningioma (eight WHO grade II meningiomas, one haemangioblastoma) and another four had been treated with conventional radiation techniques with photons prior to the proton treatment. These 13 patients were excluded from further analysis.

Regarding selection criteria, patients with large tumour remnants, those at risk of developing neurological deficits in case of tumour progression and younger patients were considered for radiation treatment. No specific cut-off values were set and the decision to refer the patient for radiotherapy lied with the neurosurgeon in charge. Patients not treated with proton radiotherapy were followed with regular magnetic resonance imaging (MRI) scans and treated at tumour relapse.

Table 1 summarises the baseline characteristics of the patient population that was finally enrolled in the study. Of the remaining 170 patients, 135 were women and 35 men (79.4% vs 20.6%). The mean age at the time of the treatment was 54.2 years (range, 22–85). Most of the patients were operated upon (*n* = 126, 74%) and the diagnosis was confirmed histologically. Forty-four patients (26%) were considered to have an unacceptably high perioperative risk or refused surgery. The diagnosis in the latter group was based on the typical clinical and radiological image. Among the patients that were operated upon, 5 underwent tumour resection Simpson grade III, 97 Simpson grade IV and 24 Simpson grade V (3, 57 and 14% respectively).

**Table 1:** Baseline characteristics of the patient population and radiotherapy protocol

Baseline characteristics	Value (%)
Total patients	170
Females	135 (79.4)
Mean age (range)	54.2 (22–85)
Tumour location	
Anterior cranial fossa	30 (17.7)
Middle cranial fossa	75 (44.1)
Posterior cranial fossa	50 (29.4)
Convexity	10 (5.9)
Centrally	5 (2.9)
No. of patients with histological diagnosis	126 (74.1)
Simpson resection grade	
Grade III	5 (3)
Grade IV	97 (57)
Grade V	24 (14)
Radiotherapy	
Post subtotal resection	84 (49.4)
At tumour relapse	42 (24.7)
Primary	44 (25.9)
Fraction (and total) doses	
6 Gy (24 Gy)	47
5 Gy (20 Gy)	107
3.5–4.5 Gy (18–29.6 Gy)	14
2 Gy (46 Gy)	2
Mean target volume – CTV (range)	12.97 cm^3^ (1–64 cm^3^)
Mean total radiation dose ±SD (range)	21.9 ± 3.7 Gy (14–46 Gy)

The majority of the tumours were located at the skull base (*n* = 155, 91%). More specifically, 30 were located in the anterior cranial fossa including sellar region (suprasellar, parasellar, intrasellar and clinoid processes), orbita/optic nerve, olfactory groove, planum sphenoidale and tuberculum sellae; 75 were located in the middle cranial fossa, including the medial sphenoid wing and cavernous sinus; finally, 50 were located in the posterior cranial fossa, including the petrous apex, clivus, cerebellopontine angle (CPA), foramen magnum and jugular foramen. Ten tumours (6%) were arising from the convexity, including the lateral sphenoid wing and superficial part of falx in proximity of or invading the superior sagittal sinus. Finally, five tumours (3%) were growing centrally, that is on the tentorium, deeper parts of the falx between the cerebral hemispheres and intraventricularly.

In a total of 70 patients (41.2%) radiological progression of tumour was documented with two or more serial MRI or computed tomography (CT) scans preoperatively or postoperatively (for residual tumour) prior to irradiation. In 21 cases (12.3%), serial radiological examinations failed to show evidence of tumour progression. In the remaining 79 patients (46.5%), the irradiation treatment was initiated without performing at least two radiological studies with sufficient time in between, either preoperatively or postoperatively (usually one preoperatively and one postoperatively to evaluate the residual tumour). Thus, possible tumour growth could not be documented in these cases.

### Irradiation technique

Stereotactic irradiation with a modified 180-MeV proton beam has been utilised at Akademiska University Hospital, Uppsala for the primary or adjuvant treatment of benign meningiomas since 1994 [12]. The proton beam delivery system used in this work with a fixed horizontal beam and passive scattering was not as flexible as modern proton facilities with gantries and intensity modulated proton beams (IMPT) using spot scanning. These limitations did in some cases compromise the dose conformality around the target and gave larger dose margins compared to current IMPT gantries.

There was a limited access of beam-time at the "The Svedberg Laboratory", where the Gustav Werner cyclotron was located. That led to considerations regarding the optimal way to exploit the available beam time of 10 weeks per year. We decided to choose a proton beam therapy only in a hypofractionated mode. The first patients were treated with 4 × 5 Gy (4 × 5.5 CGE) or 4 × 6 Gy (4 × 6.6 CGE) fractions to a total dose of 20–24 Gy (22–26.4 CGE). The treatment was given during 4 consecutive days under corticosteroid coverage. Early results were promising with few late adverse effects [13]. This treatment strategy was not changed even though the beam time availability at the laboratory was increased later on.

For precise positioning of the patient during irradiation we used a system with X-ray-opaque 2-mm cylindrical titanium markers implanted in the outer table of the skull through small skin incisions under local anaesthesia. The markers were placed two on each side of the head in the frontal and parietal regions. After the implantation, the markers were imaged on CT, with the patient’s head immobilised in a temporary fixation. The CT slices with thickness and separation of 2 mm covered the target area as well as the markers. Therefore, the same CT examination could be used to determine the coordinates of the markers and precise positioning of the target. This CT study was also used for treatment planning.

**Fig. 1:**
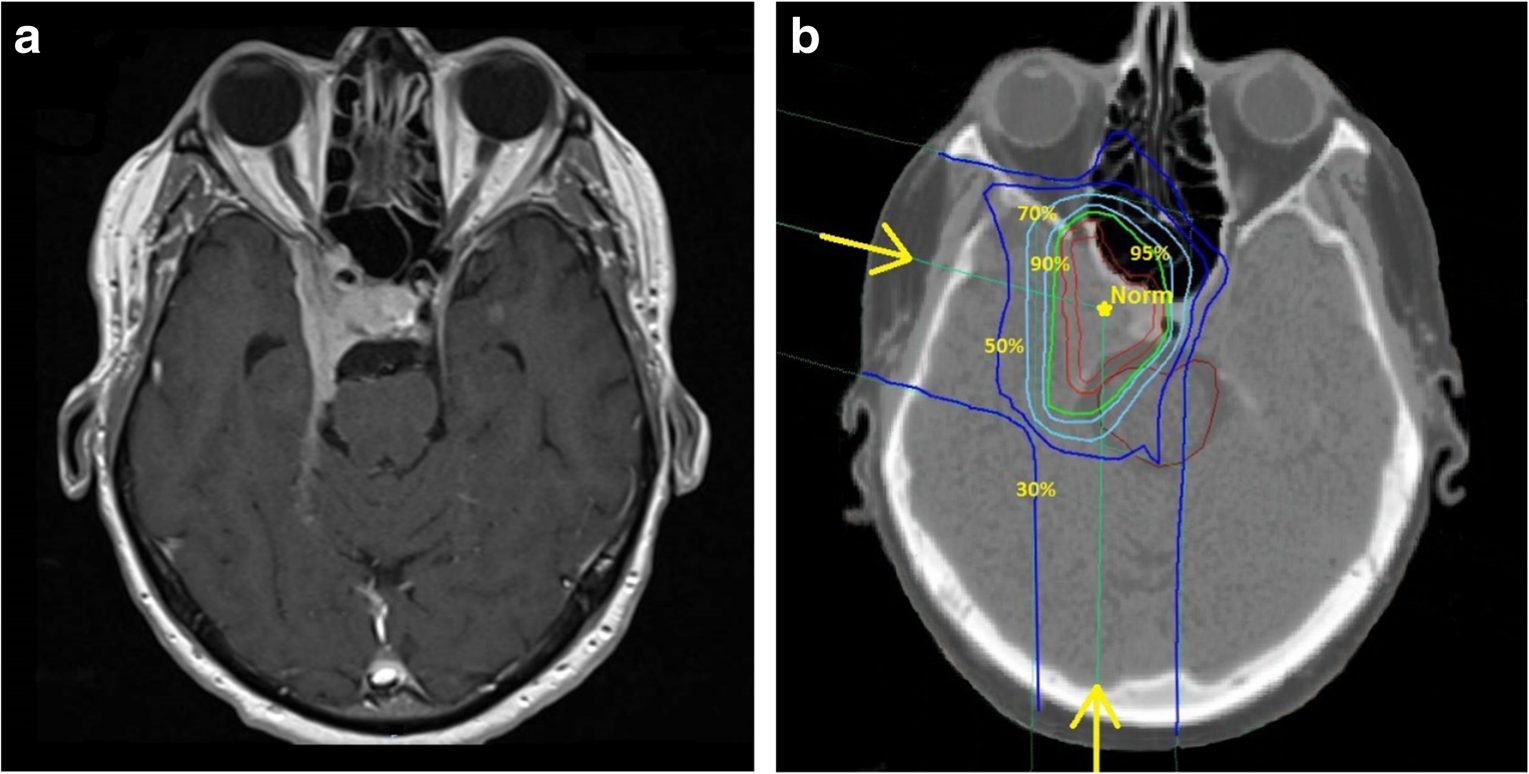
**a** MRI scan of right-sided cavernous sinus meningioma. **b** CT scan and treatment plan in section corresponding closely to the MRI scan in **a**. The dose prescribed to the target in the ICRU reference point is indicated as *Norm*. The *isodose lines* shown are 30, 50, 70, 90 and 95% of the prescribed dose

Outlining of the target and dose calculation was done in all patients using a HELAX TMS–treatment planning system (MDS Nordion, Ottawa, Canada). The clinical target volumes (CTV) were identified as being equal to the gross tumour volumes (GTV) and were defined close to the contour of the meningiomas on contrast-enhanced CT images. MRI scans were used as additional help in the target definition in many cases but only after year 2000 (Fig. 1a). The mean CTV in our material was 12.97 cm^3^ (Table 1). The planning target volume (PTV) was defined by adding a maximum margin of 5 mm around the CTV. Two or three proton-beam portals were applied (Fig. 1b). The aim was to keep all portals and their entrance doses in the same hemisphere as the targets. For centrally located targets though, it was more favourable to irradiate from both sides. All beams were given with passively scattered 180-MeV proton beams from a fixed horizontal beam using individually shaped collimators, range modifiers and modulating filters. The tumour dose was prescribed to the ICRU reference point located in a central part of the target volume (Fig. 1b) [28]. Care was taken to encircle the PTV with the 95% isodose according to the ICRU 50 protocol definition [28]. Dose limiting structures were considered to be the optic nerve, chiasm and brainstem where a maximum of 70% of the prescribed dose was allowed. In cases where the target was close to these structures, the target dose had to be compromised to meet the constraints. In accordance with our protocol, a total prescribed dose of 20 or 24 Gy was administered in four 5- or 6-Gy fractions, which corresponds approximately to an EQD2 (equivalent dose in 2-Gy fractions) of 43 Gy calculated with the linear quadratic formula [18]. The vast majority of the patients were treated according to the protocol with 5- or 6-Gy fractions (107 and 47 patients respectively). Fourteen patients were treated with various fractions between 3.5 to 4.5 Gy and the remaining two with 2-Gy fractions (Table 1).

The patients were treated in a seated position, fixed with an individually formed bite block (Optosil®) and support for the head with an individually designed helmet made of thermoplastic material (Posifix®). The first day of the treatment week was reserved for simulation of the treatment position and checking of the dose plan. Treatment was then given over 4 consecutive days with one fraction each day. In general, the patients spent 25–35 min in the treatment room per fraction. The treatment and the fixation with the bite-block were usually well tolerated. In order to avoid the risk of acute oedema around the target area, the patients were given betamethasone during the days of irradiation and in a decreasing fashion for 3 weeks thereafter.

### Follow-up procedure

After irradiation the patients were followed in a specially designed protocol with clinical examinations and radiological controls at 6 months and 1, 2, 3, 5, 7 and 10 years post treatment. The follow-up period extended until 31 December 2012, so that all patients could be followed for at least 5 years. The median follow-up time of the study was 84 months. In those cases where tumour growth or complications related to the irradiation treatment were noticed, appropriate therapies were initiated (re-operation, second session of radiotherapy or radiosurgery).

### Statistics

All statistical analyses were performed with the Statistica® software (Stat Soft, Tulsa, OK, USA). Kaplan Meier curves were used for estimation of progression-free survival (PFS) rates. Multiple logistic regression models including odds ratios were applied for analysis of possible significant prognostic factors predicting higher risk for tumour relapse and radiation-related complications. A *p* value <0.05 and confidence intervals (CIs) of 95% were considered statistically significant.

## Results

The overall mortality rate during the period of the study (1994–2012) was 13.5% (23/170 patients). The cause of death was identified in 17 cases with cancer being the most common. In three cases death could be related to the treated meningioma resulting in a disease-specific mortality of 1.7%. In one case the tumour increased in size 3 years after irradiation but no further treatment was initiated due to the patient’s age (81 at the time of progression). The patient died 2 years later. In another case the tumour recurred 3 years after initial treatment with resection plus proton radiotherapy and the biopsy after reoperation showed transformation to WHO grade II tumour. The patient underwent additional radiotherapy with photons and died 5 years later from further tumour progression. Finally, the third case was a patient with neurofibromatosis type 2, who died due to refractory status epilepticus after showing tumour growth shortly post irradiation. However, this patient had multiple other tumours as well and the irradiated meningioma was located at the clivus, thus making it improbable that the meningioma was the actual cause of status epilepticus and death.

### Effectiveness

**Fig. 2:**
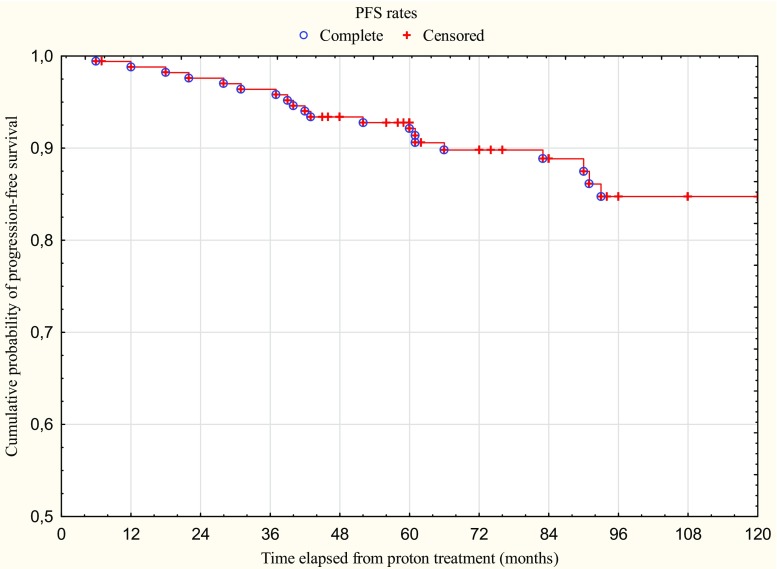
Kaplan Meier curve showing the progression-free survival rates at different time intervals. *Complete* tumour progression, *Censored* dead or lost in follow-up)

Twenty patients showed tumour progression during the follow-up period, the majority within 5 years from proton treatment (13/20). In 12 cases (60%) radiological progression of the tumour had been noticed even before treatment. The actuarial 5- and 10-year PFS rates that occurred after Kaplan Meier analysis were 93% and 85% (Fig. 2).

Multiple logistic regression models including odds ratios were used to evaluate possible prognostic factors that significantly influenced the treatment outcome and predicted lower risk for tumour progression. The factors that were examined were age, sex, tumour localisation (anterior vs middle vs posterior middle fossa vs miscellaneous), resection grade according to Simpson scale, i.e. subtotal resection (grade 3 and 4) vs no resection (grade 5 and no surgery) and, finally, target volume (CTV).

The results of the multivariate analysis are presented in Tables 2 and 3. Factors that were significantly associated with lower risk of tumour progression were higher age (OR = 1.06, *p* = 0.01) and tumour localisation in the middle cranial fossa (OR = 8.55, *p* = 0.04) (Table 2). Among the categorical variables (sex, tumour localisation and STR), males, miscellaneous tumour localisation, i.e. other than the skull base, and no resection were used as references, as shown in Table 3.

**Table 2:** Multiple logistic regression model estimating probabilities for stable tumour post irradiation in different clinical parameters

	Tumour progression - odds ratios					
	Distribution: BINOMIAL, Link function: LOGIT					
	Modelled probability that tumour progression = no					
Effect	Level of effect	Column	Odds ratio	Lower CL 96.0%	Upper CL 95.0%	*p*
Intercept		1				
Age of treatment		2	1.061573	1.014751	1.11055	0.009425
CTV		3	1.056251	0.989277	1.12776	0.101549
Sex	Female	4	2.376454	0.753013	7.49997	0.139892
Tumour localisation	Anterior	5	2.555279	0.565480	11.54674	0.567192
Tumour localisation	Middle	6	8.660980	1.724616	42.39742	0.043157
Tumour localisation	Posterior	7	5.297341	1.136567	24.68998	0.288145
STR	Yes	8	0.517534	0.159137	1.68308	0.273647

**Table 3:** The results from the multivariate analysis of potential prognosticators for tumour progression including the categories that were used as references in the different variables

Variable	OR, 95% CI	*p* value
Age	1.06 (1.01–1.11)	0.009
CTV	1.06 (0.99–1.13)	0.10
Sex		
Male	*R*	*R*
Female	2.38 (0.75–7.50)	0.14
Localisation		
Miscellaneous	*R*	*R*
Anterior fossa	2.56 (0.57–11.6)	0.57
Middle fossa	8.55 (1.72–42.4)	0.04
Posterior fossa	5.30 (0.16–1.68)	0.29
STR		
No	*R*	*R*
Yes	0.52 (0.16–1.68)	0.27

*OR* odds ratio, *R* reference category, *STR* subtotal resection, *CTV* clinical target volume, *CI* confidence interval, *miscellaneous* other than the skull base

### Complications

Complications after treatment were divided into four types and were noticed in a total of 16 patients. The four types were pituitary insufficiency, radiation necrosis, visual impairment and expansive tumour cyst. One patient developed two different complications (pituitary insufficiency and radiation necrosis).

The number of patients in whom significant dose of irradiation (defined as >50% of the mean dose of proton beam) passed “through” the pituitary was 81. Six of those patients (7.4%) developed pituitary insufficiency requiring substitution therapy months to years after irradiation. All these tumours were located in proximity to the sella (four supra/parasellar, two at clivus).

Five patients (2.9%) showed signs of radiation necrosis at different times during the radiological follow-up. Only one had a “clinically significant” necrosis requiring temporal lobe resection due to mass effect; the rest were noticed on MRIs only and were totally asymptomatic.

Parts of the optic apparatus (optic nerves and chiasma) were fully or partially included in the radiation fields in 112 cases. Five patients (4.4%) showed different types of visual impairment, either visual deterioration or visual fields deficits. An explanation to the relatively high incidence of visual impairment can be the fact that the delineation of the optic nerves largely was based on CT imaging alone. MRI would probably give a more adequate definition of these structures.

Finally, one patient (0.5%) was found to have an expansive tumour cyst of unknown nature on MRI control 6 months after treatment. The cyst was asymptomatic at the time of diagnosis, so no additional treatment was required and resolved spontaneously at 1 year control.

The same parameters as for tumour progression (age, sex, tumour localisation, resection grade and CTV) were assessed in a new multiple logistic regression model. No patients with tumour localisation other than the skull base suffered from complications related to the irradiation. Due to complete separation that affected the results, tumour localisation outside the skull base was excluded from the logistic regression analysis. Localisation in the anterior cranial fossa was the only factor significantly associated with the development of complications post irradiation, as shown in Table 4 (OR = 4.77, *p* = 0.01). Table 5 shows the parameters among the categorical variables that were used as references (males, localisation in the posterior cranial fossa and no STR).

**Table 4:** Multiple logistic regression model estimating probabilities for the development of complications post irradiation in different clinical parameters

	Complication post radiation - odds ratios					
	Distribution: BINOMIAL, Link function: LOIT					
	Modelled probability that complication post radiation = yes					
Effect	Level of Effect	Column	Odds Ratio	Lower CL 95.0%	Upper CL 95.0%	*p*
Intercept		1				
Age at treatment		2	1.006828	0.961315	1.05450	0.773111
CTV		3	1.022044	0.981899	1.06383	0.286191
Sex	Female	4	0.316.452	0.096987	1.03253	0.056533
STR	Yes	5	1.663447	0.493873	5.60277	0.411453
Tumour localisation	Anterior	6	4.777198	1.102311	20.70343	0.014016
Tumour localisation	Middle	7	1.139274	0.2717%	4.77544	0.272950

**Table 5:** The results from the multivariate analysis of potential prognosticators for development of complications including the categories that were used as references in the different variables

Variable	OR, 95% CI	*p* value
Age	1.01 (0.96–1.05)	0.77
CTV	1.02 (0.98–1.06)	0.27
Sex		
Male	*R*	*R*
Female	0.32 (0.10–1.03)	0.06
Localisation		
Posterior fossa	*R*	*R*
Anterior fossa	4.78 (1.10–20.7)	0.01
Middle fossa	1.14 (0.27–4.78)	0.27
STR		
No	*R*	*R*
Yes	1.66 (0.49–5.60)	0.41

*OR* odds ratio, *R* reference category, *STR* subtotal resection, *CTV* clinical target volume, *CI* confidence interval

## Discussion

The aim of this study was to evaluate proton irradiation technique in the treatment of benign meningiomas focusing on tumour growth control rates and possible complications and to compare the results with other more frequently used methods of irradiation. For that purpose 170 patients with benign meningiomas who were treated consecutively with protons were enrolled and followed-up for at least 5 years after irradiation (unless the tumour recurred or the patient died before). One patient was completely lost from follow-up. Nineteen of those patients were first included in a pilot study that was published in 1999 [13]. The patients were treated with a total dose of 24 Gy given in four 6-Gy fractions and were followed up for three years. No patient showed signs of tumour progression and in one patient slight deterioration of the clinical status was noticed 6 months after treatment but the situation improved at 12 months.

The 5- and 10-year PFS rate in the current study were 93% and 85% respectively which is satisfactory, especially with regard to the large tumour volumes (mean target volume 13 cm^3^), and comparable with the results from other studies [15, 26, 41, 42]. No deaths could be directly attributable to the radiation treatment. Complications were seen in 9.4% of the patients but the majority were either asymptomatic or treated pharmacologically with good effect (i.e. hypopituitarism).

More serious complications were also noticed. One patient became blind after irradiation. In that case the tumour was located on the optic nerve and despite the fact that smaller fraction doses were used (2-Gy fractions up to 46 Gy) the blindness in the ipsilateral eye was inevitable. Four patients developed milder visual deficits (two cases of deterioration of visual acuity and two of visual fields). In the cases with visual field deficits the tumour was located on the optic nerve and the patients had slight deficits already before the irradiation which worsened afterwards. In one case with deterioration of visual acuity, the treated meningioma was located in the medial sphenoid wing and the problem was identified to be the direction of one of the two fields that included the optic nerve and artery. In the other case the tumour was located in the medial sphenoid wing and the treatment was given in 4 × 6-Gy fractions to a total dose of 24 Gy including parts of the optic pathways. Visus deterioration might have been avoided with lower fraction doses. Finally, one patient with cavernous sinus meningioma developed radiation necrosis in the ipsilateral temporal lobe that required resection due to mass effect. Retrospective analysis of the treatment protocol for this patient revealed that the meningioma was treated with 4 × 6-Gy fractions to a total of 24 Gy through two portals. For two proton-beam portals the entrance dose lies in the range of 30-50% of the target dose, i.e. approximately 2 Gy × 4 or 2.5 Gy × 4. In our opinion, these dose levels are well below the risk for temporal lobe necrosis. The beam delivery technique used for these patients was passive scattering, which means that the conformality was not as good as can be achieved today with intensity-modulated proton beams (spot scanning). The larger dose margin around the target given with passive scattering could explain the development of temporal lobe necrosis.

The large number of patients included and the long follow-up period are the main strengths of this study. Regarding weaknesses, the study comes from a single centre and there is some inhomogeneity in the fraction doses as two patients were treated with 2-Gy fractions which is conventional fractionation and 14 with doses between 3.5–4.5 Gy (though the latter doses can be considered hypofractionated). In addition, 46 patients were lacking histological confirmation of their tumours and were treated for presumed grade I meningiomas. Finally, the pathological classification of the tumours was made based on the WHO guidelines from 2003. The revised guidelines that came out in 2007 changed the classification criteria in a way that some tumours which were classified as grade I based on the previous system now became grade II; that could possibly affect our material since some grade I tumours could actually be grade II, had they been classified with the 2007 system [22].

Radiotherapy/radiosurgery primary or secondary after surgery is nowadays a well-established treatment method for residual, recurrent or primarily unresectable meningiomas. Several modalities are available for that purpose; for example, external photon beam radiation therapy with conventional fractionation or intensity-modulated radiation therapy (IMRT), volumetric arc therapy (VMAT), stereotactic radiosurgery with photons (Gamma Knife-LINAC) either in a single fraction or with hypo-fractionation, high-energy protons or photon/proton combinations. Besides surgery and radiotherapy, there are currently no efficient medical options in the treatment of meningiomas. There are examples from palliative treatments, e.g. somatostatine analogues, however with negligible effect. Chemotherapy has not been proven to give any satisfactory responses [3, 23].

Minniti et al. [26] reviewed studies about treatment of skull base meningiomas with conventionally fractionated radiotherapy reporting 5 years PFS rates of 76–95% (90% on average). Complications reported in these series were radiation necrosis (rare), from optic pathways (0–3%), other cranial nerves deficits (1–3%) and hypopituitarism (5%). IMRT series were also reviewed in the same article. The largest series by Milker-Zabel et al. [25] included 94 patients, some with atypical or malignant meningiomas, treated with doses of 57.6 Gy with a median follow-up of 4.4 years. The reported tumour control rate was 93.6% at 5 years with neurological worsening occurring in 4% of the patients. Other smaller series reported tumour-control rates of 93–97% but with fewer patients and shorter follow-up [36, 44].

Stereotactic radiosurgery with Gamma Knife (GKS) or Linear accelerators (LINACs) has been widely used in the treatment of meningiomas with several studies published in the literature with varying numbers of patients and follow-up duration. The GKS experience shows that this technique is more suitable for smaller tumours (less than 10 cm^3^) with well-defined margins and sufficient distance from critical structures [1]. In a recently published large multicentre study Santacroce et al. [37] reported on 4,565 patients with 5,300 benign meningiomas treated with GKS. The mean tumour volume was 4.8 cm^3^, median dose to tumour margin was 14 Gy and the median follow-up 63 months. The 5-year PFS rate was 95.2% and the permanent morbidity rate was estimated at 6.5%. Starke et al. [42] reported a 5-year PFS rate of 96% in a series of 255 patients treated with GKS for skull base meningiomas where the mean tumour volume prior to treatment was 5 cm^3^ and the mean follow-up 6.5 years. In another study from the same group, 75 patients with large skull base meningiomas, defined as volume >8 cm^3^ (diameter >2.5 cm), were treated with GKS with a mean follow-up of 6.5 years [41]. The 5-year PFS rate in this study declined to 88% (compared to 96% in the previous study) with deterioration of neurological function in 17% of the patients. Pollock et al. [33] treated 416 patients harbouring benign meningiomas (252 image-defined and 164 histologically confirmed) with single fraction GKS. The mean tumour volume was 7.3 cm^3^, the mean dose 16 Gy and the median follow-up 60 months. The reported 5-year PFS was 96% and permanent radiation-related complications were seen in 11% of the patients. Kimball et al. [20] reported on 55 patients treated with LINAC radiosurgery for cavernous sinus meningiomas (median volume 5.9 cm^3^, median follow-up 50 months) where the actuarial 5-year local tumour control rate was 100% with one patient developing new cranial nerve (CN) deficit and one patient showing worsening of previously existing CN deficit.

Protons have been used in the treatment of meningiomas to a lesser extent compared to GKS and in varying settings (with conventional fractionation, hypofractionation, single-session SRS or combined with photons). One of the earliest published studies by Wenkel et al. [46] reported on 46 patients with benign meningiomas treated with combined photon and proton beam therapy at the Massachusetts General Hospital in Boston, USA. Recurrence-free rate at 5 and 10 years were 100% and 88% respectively but severe complications were also seen with one death case from brain stem necrosis and eight patients developing severe long-term toxicity of radiotherapy. Noel et al. [30] reported a 4-year local control rate of 87.5 ± 12% on 17 patients (5 with atypical meningiomas, 12 with benign but recurrent or rapidly progressive) treated with highly conformal photon and proton beam. Weber et al. [45] utilised proton radiotherapy with conventional fractionation for the treatment of intracranial meningiomas in 16 patients (median gross tumour volume 17.5 cm^3^, median dose 56 Gy[RBE-corrected] at fractions of 1.8–2 Gy[RBE-corrected] and median follow-up 34.1 months) and reported a 3-year PFS of 91.7%, with three patients developing radiation-related complications. Hypofractionated protocols have also been used, with Vernimmen et al. [47] reporting on 23 patients, 5 of whom were treated with stereotactic radiotherapy (≥16 fractions) and 18 with hypofractionated radiotherapy. The mean target volume in the latter group was 15.6 cm^3^ and the mean clinical and radiological follow-up periods were 40 and 31 months respectively. Sixteen out of 18 (88.8%) patients in that group showed good clinical status and radiological tumour control at the end of the follow-up period, 2 out of 18 suffered permanent neurological deficits. Finally, in a more recent study, Halasz et al. [15] reported the first series of proton stereotactic radiosurgery for the treatment of meningiomas. Fifty patients were included in the study with median tumour volume of 2.1 cm^3^, median prescribed dose 13 Gy[RBE-corrected] and median follow-up 32 months. The 3-year actuarial control rate was 94% and the rate of potential permanent adverse effects of radiosurgery was 5.9%.

The development of radiosurgery and stereotactic radiotherapy, including proton-beam therapy, offers a more precise treatment compared to older conformal 2D and 3D techniques with photons. With improved fixation techniques, more sophisticated analysis with CT and MRI for target definition and treatment with protons, a reduction is to be expected in late reactions in surrounding normal tissue both in frequency and intensity.

The results of this study suggest that protons are an equivalent alternative to other more frequently used methods of radiation in the treatment of meningiomas with high rates of long-term tumour growth control. The physical and dosimetric features of proton beams (no dose delivered “downstream” of the target and high conformality even to irregularly shaped targets) make them particularly interesting for patients with relatively large tumours, since the mean target volume in our material (almost 13 cm^3^) was clearly larger than in many other similar series in the literature. The somewhat problematic incidence of radiation-related complications may reflect some difficulties in defining the target based on CT images alone (which was exclusively used in the first patients of the series, MRI images as support became available after year 2000) but also the larger target volumes in our material.
